# Experimental Study on the Mechanism and Law of Low-Salinity Water Flooding for Enhanced Oil Recovery in Tight Sandstone Reservoirs

**DOI:** 10.1021/acsomega.3c07960

**Published:** 2024-03-05

**Authors:** Pingtian Fan, Yuetian Liu, Yuting He, Yuanping Hu, Leihui Chao, Yapeng Wang, Lang Liu, Jingpeng Li

**Affiliations:** † State Key Laboratory of Petroleum Resources and Prospecting, China University of Petroleum (Beijing), Beijing 102249, China; ‡ Nanniwan Oil Production Plant, Yanchang Oilfield Co., Ltd., Yan’an 716000, China

## Abstract

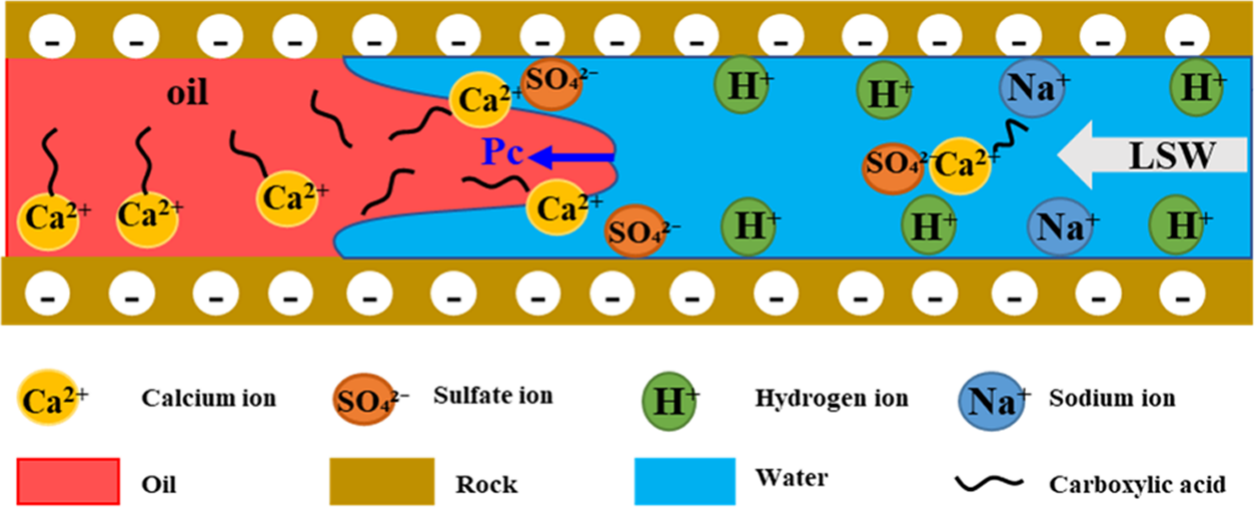

Currently, research surrounding low-salinity water flooding predominantly focuses on medium- to high-permeability sandstone reservoirs. Nevertheless, further investigation is necessary to implement this technique with regard to tight sandstone reservoirs. The present study comprises a series of experiments conducted on the crude oil and core of the Ordos Chang 6 reservoir to investigate the influence of ionic composition on low-salinity water flooding in tight oil reservoirs. The change in wettability on the rock surface was analyzed by using the contact angle experiment. The change in recovery rate was analyzed using a core displacement experiment. The reaction between rock fluids was analyzed using an ion chromatography experiment. Additionally, a nuclear magnetic resonance (NMR) experiment was used to analyze the mobilization law of crude oil and the change in wettability on the scale of the rock core. This led to a comprehensive discussion of the law and mechanism of enhancing the recovery rate via low-salinity water flooding from various perspectives. Experiments show that low-salinity water flooding is an effective technique for enhancing recovery in tight sandstone reservoirs. Altering the ionic composition of injected water can improve the water wettability of the rock surface and enhance recovery. Decreasing the mass concentration of Ca^2+^ or increasing the mass concentration of SO_4_^2–^ can prompt the ion-exchange reaction on the rock surface and detachment of polar components from the surface. Consequently, the wettability of the rock surface strengthens, augmenting the recovery process. Nuclear magnetic resonance experiments evidence that low-salinity water injection, with ion adjustment, significantly alters the interactions between the rock and fluid in tight sandstone reservoirs. As a result, the *T*_2_ signal amplitude decreases significantly, residual oil saturation reduces considerably, and the hydrophilic nature of the rock surface increases.

## 1 Introduction

With the rising global energy demand, the sustainable development of low-permeability and ultralow-permeability reservoirs is of great importance.^1−3^ In recent decades, low-permeability and extra-low-permeability sandstone reservoirs in the Ordos Basin have been mainly developed by water injection, with generally low recovery rates.^4^ As one of the lowest cost technologies to improve recovery, low-salinity water flooding has been verified by a large number of experiments.^5−8^ It has been gradually developed into a new technology for enhanced recovery by adjusting the ionic composition of injected water according to the mineral and fluid characteristics of the reservoir.^9,10^ Numerous studies have demonstrated that low-salinity water flooding can enhance recovery and have provided a variety of understanding of the recovery enhancement mechanism.^11−17^

It has been suggested by numerous researchers that the low-salinity water flooding effect is due to the alteration of wettability. The change in wettability leads to the release of crude oil adsorbed on the rock surface, enhancing recovery.^18−20^ Alhuraishawy et al.^21^ found that recovery rates increased as the concentration of NaCl and CaCl_2_ saline solutions decreased. NaCl showed higher recovery rates at a certain salinity level than CaCl_2_ in the core displacement, and the pH of the produced water shifts toward alkalinity for both substances. The transportation of clay and small mineral particles leads to the redistribution of flow channels, creating new flow paths, thus enhancing displacement and sweep efficiency. Al-Saedi and Flori^22^ found that the alkaline components in crude oil primarily adsorb onto the surface of sandstone minerals through hydrogen bonds. Injecting low-salinity water raises the pH in the formation water, resulting in the breakage of hydrogen bonds and the subsequent desorption of the alkaline components adsorbed on the mineral surfaces, thus ultimately altering the wettability of the mineral surface. Lager et al.^23^ concluded that the increase in pH is a result of low-salinity water flooding, not the cause, and that ion exchange is the real factor. The acidic components in crude oil are mainly adsorbed on the sandstone surface through the polyvalent cations (e.g., Ca^2+^) in the formation water. When low-salinity water is injected, the cations in the water replace cations on the surface of the rock (e.g., Ca^2+^). This results in the desorption of organic complexes and a change in the wettability of the sandstone surface. This phenomenon is known as the multicomponent ion exchange (MIE) mechanism.

Previous studies on low-salinity water flooding mainly focused on the effect of conventional medium and high-permeability reservoirs and proposed the mechanism of wettability change, particle transport, and multicomponent ion exchange. Low-salinity water flooding can result in the transportation of clay particles that block pore spaces, thus reducing reservoir permeability and recovery. Moreover, low pore permeability leads to the rapid advancement of the water line during the flooding process, the rapid increase of water content in the produced fluid after seeing water, and a shorter water-containing oil production period. Therefore, it is necessary to conduct an in-depth study of the corresponding mechanism and law of action to determine whether low-salinity water flooding can be applied to tight sandstone reservoirs. In this study, the formation water, crude oil, and core of the Chang 6 reservoir are taken as research objects. The contact angle, core displacement, and nuclear magnetic resonance experiments were conducted to investigate low-salinity water flooding in tight sandstone reservoirs and analyze its effects and laws of action. These studies establish the foundation for the practical application of low-salinity water flooding.

## 2 Materials and Methods

### 2.1 Materials

#### 2.1.1 Crude Oil

The experimental oil used was crude oil from the Chang 6 reservoir group, with relevant parameters shown in Table 1. Table 1 shows the physicochemical characteristics of the crude oil samples used in the study, including total acid number (TAN), total base number (TBN), and SARA volume fraction. The TAN and TBN were also measured through potentiometric titration with KOH according to ASTM D664^24^ and ASTM 2896,^25^ respectively. Crude oil samples were centrifuged before being used in the experiments to remove any possible emulsions and solids.

**Table 1:** Parameters of the Crude Oil

				volume fraction of components (%)				
viscosity (mPa·s)	density (g·cm^–3^)	TAN (mgKOH·g^–1^)	TBN (mgKOH·g^–1^)	saturated hydrocarbons	aromatics	resin	asphaltenes	total
16	0.821	0.45	0.73	73.51	21.53	2.33	2.63	100.00

#### 2.1.2 Aqueous Solutions

Table 2 shows the ionic composition and solubility product *K*_sp_ of the aqueous solution in the experiment. The *K*_sp_ value for Ca^2+^ and SO_4_^2–^ ions in this experiment remains under the *K*_sp_ of CaSO_4_ in pure water, which is 4.93 × 10^–5^ (25 °C).^26,27^ Additionally, the presence of multiple ions in the solution allows for increased solubility of CaSO_4_, which can avoid the effects of CaSO_4_ precipitation during the experiment.^28^ Chemicals such as CaCl_2_, MgCl_2_, KCl, NaCl, and Na_2_SO_4_ (Aladdin) were weighed accurately and then dissolved in distilled water (prepared in the laboratory) to create the solutions. Stirring for 1 h ensured the solutes dissolved uniformly, followed by filtering through a 1 μm diameter filter membrane to remove any potential impurities. The experimental aqueous solutions were prepared and used immediately to minimize the impact of the ambient contaminants. In this experiment, the formation water (FW) salinity was 38,149.92 mg/L. The low-salinity water (LSW)’s salinity was 5535.537 mg/L. Since monovalent ions (Na^+^, Cl^–^) have a weaker effect on the interactions between rock and fluid, as compared to divalent ions, we adjusted the mass concentration of Na^+^ and Cl^–^ in the solution to ensure the concordance of salinity.^29,30^ LSW-0.5Ca^2+^ denotes the solution in which the salinity remains constant, but the mass concentration of Ca^2+^ is reduced by half. To ensure salinity conservation, the Na^+^ mass is increased accordingly. Similarly, LSW-0.5Ca^2+^-2SO_4_^2–^ denotes the solution that maintains a constant salinity. This is achieved by doubling the mass concentration of SO_4_^2–^ based on the ionic composition of LSW-0.5Ca^2+^. To ensure conservation of the salinity in the solution, the increased mass of SO_4_^2–^ ions is balanced by a reduction in the mass of Cl^–^.

**Table 2:** Ionic Composition and Solubility Product *K*_sp_ of the Aqueous Solution in the Experiment

solutions	Na^+^ (mg/L)	K^+^ (mg/L)	Mg^2+^ (mg/L)	Ca^2+^ (mg/L)	Cl^–^ (mg/L)	SO_4_^2–^ (mg/L)	salinity (mg/L)	*K*_spCaSO_4__ (mol^2^/L^2^)
FW	7952.25	0	0	8022.43	21692.1	302.915	38149.92	6.31 × 10^–4^
LSW	851.124	55.5915	338.442	682.681	3490.644	117.054	5535.537	2.08 × 10^–5^
LSW-0.2Ca^2+^	1150.68	55.5915	338.442	136.536	3707.218	117.054	5535.537	4.15 × 10^–6^
LSW-0.5Ca^2+^	1057.105	55.5915	338.442	341.340	3626.31	117.054	5535.537	1.04 × 10^–5^
LSW-0.8Ca^2+^	933.517	55.5915	338.442	546.144	3544.786	117.054	5535.537	1.66 × 10^–5^
LSW-0.5Ca^2+^-2SO_4_^2–^	1057.105	55.5915	338.442	341.340	3509.256	234.108	5535.537	2.08 × 10^–5^
LSW-0.5Ca^2+^-3SO_4_^2–^	1057.105	55.5915	338.442	341.340	3392.202	351.162	5535.537	3.11 × 10^–5^
LSW-0.5Ca^2+^-4SO_4_^2–^	1057.105	55.5915	338.442	341.340	3275.148	468.216	5535.537	4.15 × 10^–5^

#### 2.1.3 Rock Cores

**Figure 1:**
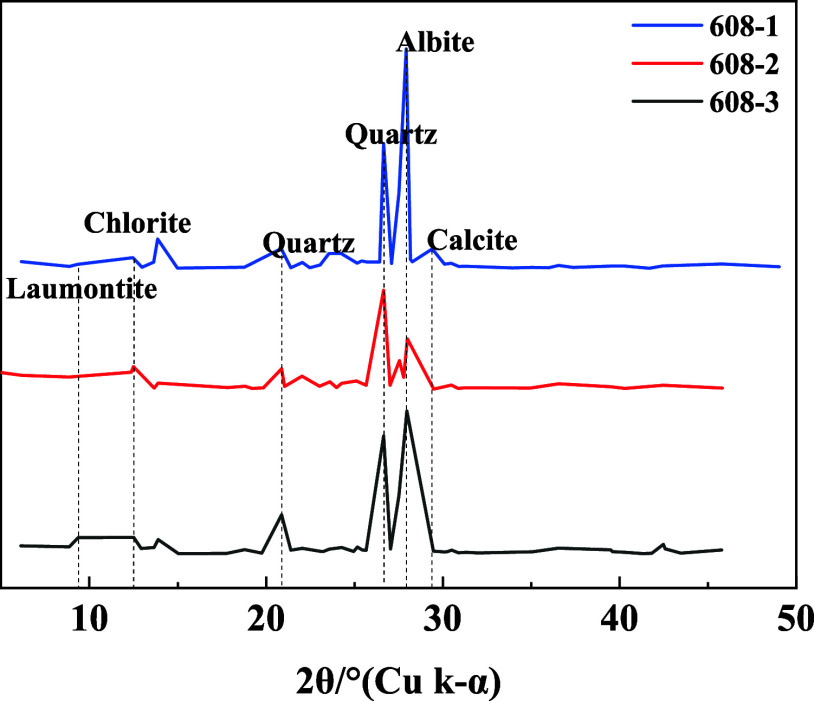
X-ray diffraction results of the experimental core samples.

Wettability determination, core flooding, and nuclear magnetic resonance experiments were carried out on core samples extracted from the tight sandstone of the Chang 6 reservoir located in the Ordos Basin. Figure 1 presents the X-ray diffraction results of the three rock samples. To determine the mass fractions of several components, the 2θ intensity data from the samples are compared with known mineral standard 2θ intensity data. The calculated mean mass fractions of each constituent are plagioclase feldspar (38.5%), quartz (22.3%), clay minerals (13.9%), potassium feldspar (12.3%), and other constituents, namely, zeolite, gypsum, siderite, and calcite, make up 7.2, 2.3, 1.4, and 1.1%, respectively. The mass fractions of the clay minerals were also analyzed, showing chlorite (62%), kaolinite (14%), illite-smectite mixed layers (13%), and illite (11%).

### 2.2 Cores Preparation

The core samples were dried in a vacuum drying oven at 120 °C for 2 days, measuring the net weight every 12 h until the net weight of the cores stabilized. Nitrogen porosity measurements (PMI-100 Helium Porosity Measurement Instrument, from Beijing Yineng Petroleum Technology Co., Ltd.) and gas permeability tests (ULP-613 Ultra-Low-Permeability Core Gas Permeability Automatic Tester) were conducted to measure the porosity and gas permeability of each core. After being dried, the cores were immersed in formation water for vacuum saturation and preaging for 2 days to ensure complete saturation of the pore spaces with formation water. Following that, the cores were placed in the core holder, subjected to a confining pressure of 30 MPa, and displaced with formation water at a constant flow rate of 0.1 mL/min. The liquid permeability was calculated during this process. Crude oil was consistently injected into the cores at 10 MPa until no more water was produced. The initial oil saturation and bound water saturation were calculated, followed by aging at 90 °C for 3 weeks.^31^ The sandstone core physical property data are presented in Table 3, with an average permeability of approximately 0.027 mD.

**Table 3:** Physical Properties of the Experimental Core

core ID	liquid permeability (mD)	gas permeability (mD)	porosity (%)	reservoir classification
608–1	0.02785	0.56–0.65	8.0496	tight
608–2	0.02631		7.9022	tight
608–3	0.02574		8.0667	tight
608–4	0.02742		7.9214	tight
608–5	0.02889		7.9853	tight
608–6	0.02734		7.9476	tight
608–7	0.02711		7.9231	tight
608–8	0.02692		7.9823	tight
608–9	0.02783		7.9427	tight

### 2.3 Contact Angle Measurement

**Figure 2:**
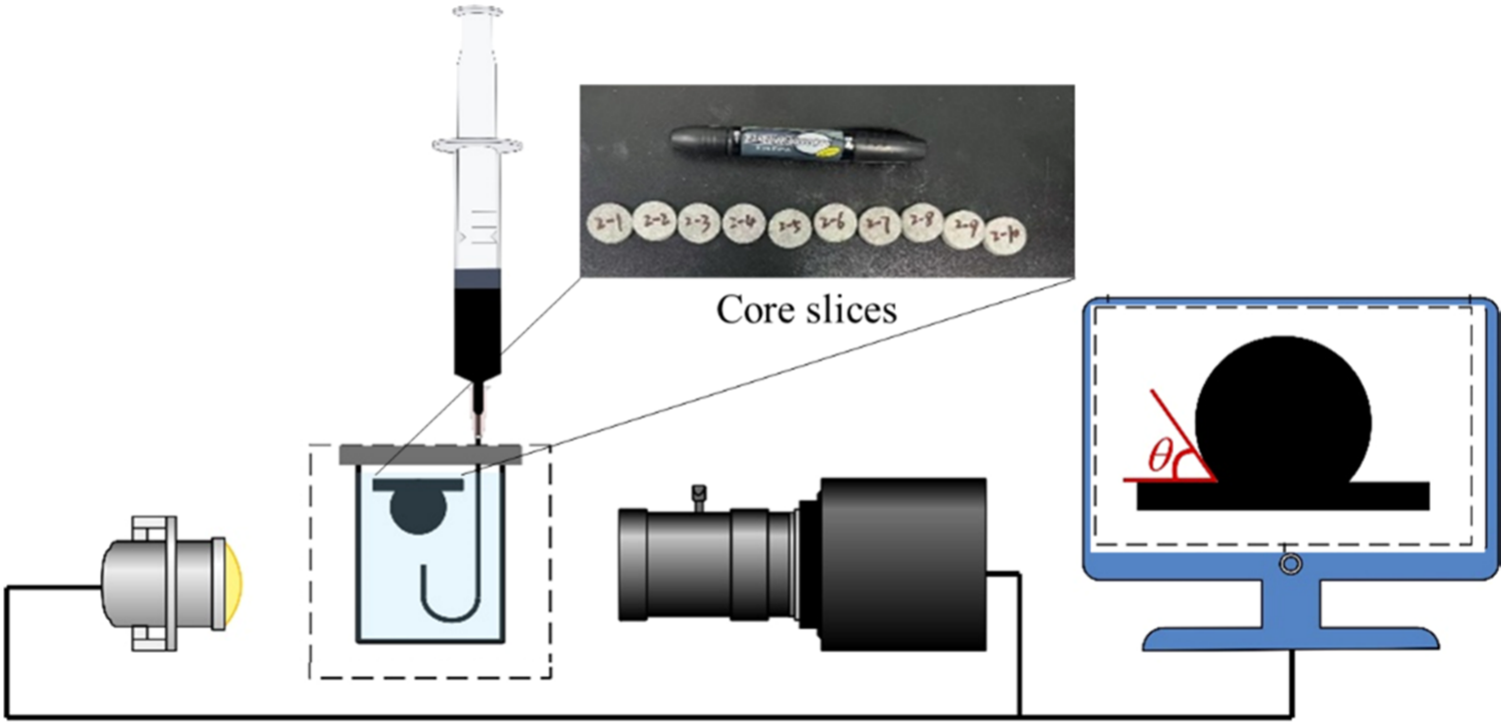
Contact angle measuring device.

The contact angle measuring device (DC-200, Sindin) and core slices are shown in Figure 2. The cores were cut into thin slices of 3 to 5 mm thickness and placed in conical flasks filled with formation water and vacuumed until no bubbles emerged from the surface of the slices. Subsequently, the saturated cores were immersed in crude oil, placed in a thermostat, and aged for 45 days before conducting the experiments at 90 °C.^31,32^ The samples were later washed with *n*-heptane and finally dried in an oven for 1 day. The core slices were immersed in an aqueous solution. The u-shaped needle was employed to drop 15 μL of oil on the surface of the core slices, and a dynamic study of the contact angle was conducted at 30 °C.

### 2.4 Core Displacement Experiment

**Figure 3:**
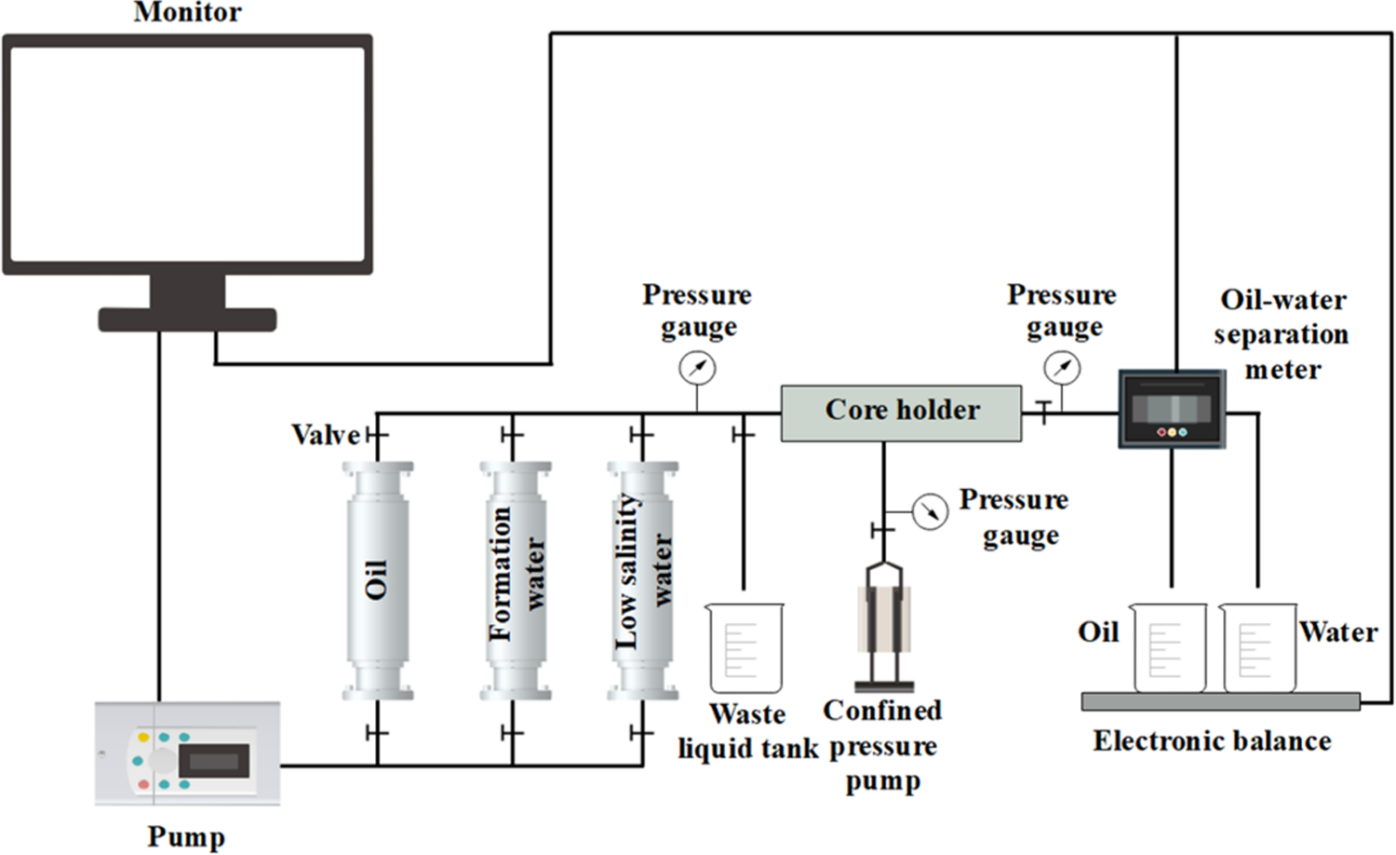
Displacement experiment analysis system.

The displacement experiment analysis system is shown in Figure 3. The displacement experiment system consists of a precision injection pump (Teledyne ISCO 500x), an intermediate vessel (Halan Oil Scientific Instrument Co., Ltd.), and a core holder (Halan Oil Scientific Instrument Co., ltd.). The rock cores were positioned in the core holder and subjected to displacement experiments at 30 °C and a confining pressure of 30 MPa. Different aqueous solutions with various ionic compositions were injected at a flow rate of 0.1 mL/min. The amount of oil and liquid produced was recorded in real time by utilizing a high-precision oil–water separation meter and electronic balance (Mettler XPR204S/AC). The formation water was initially used for displacement. After the rock core outlet pressure stabilized and the water cut reached 98% or higher, the injection fluid was switched to continue the subsequent displacement process.

### 2.5 Ion Chromatography Experiment

In this experiment, various ion compositions of the injection water are utilized to investigate water–rock reactions. The rock samples are first ground into powder and sieved through a 320-mesh sieve to ensure a consistent particle size. Subsequently, 10 g of the rock powder is mixed with 20 mL of injection water containing different ion compositions. The mixture is thoroughly stirred for 30 min at a temperature of 30 °C and then allowed to settle for 2 h. After the settling period, the supernatant is collected for further analysis. The pH value of the supernatant is measured to assess the acidity or alkalinity of the solution at 25 °C. The output solution was diluted 20 times with distilled water (prepared in the laboratory), filtered through a 0.2 μm membrane, injected into the sample chamber, and tested in ion chromatography experiments at 25 °C. To determine the ion composition present in the supernatant, inductively coupled plasma-optical emission spectroscopy (ICP-OES) is employed. Specifically, a PerkinElmer 2000D ICP-OES instrument is used for the analysis.

### 2.6 NMR Experiment

**Figure 4:**
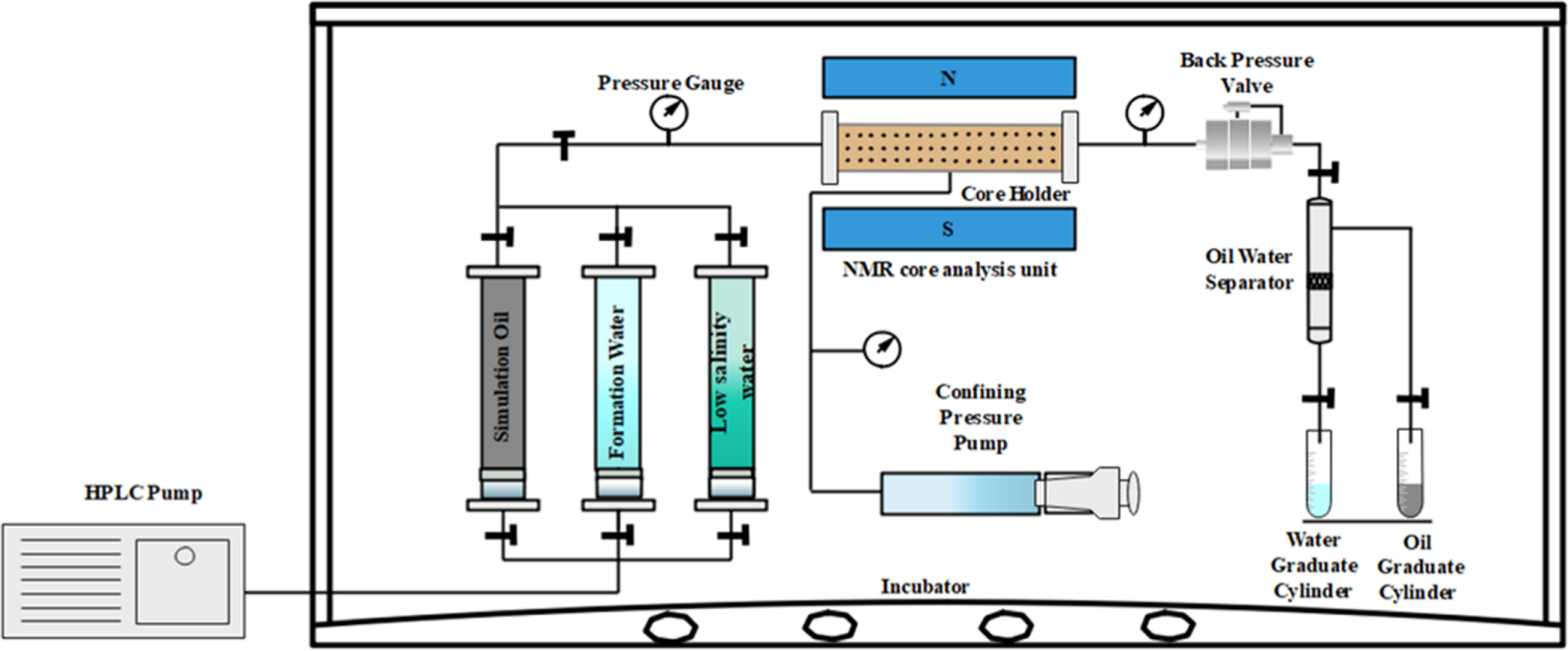
Low-field nuclear magnetic resonance rock core analysis system.

Nuclear magnetic resonance experiments were conducted using a low-field nuclear magnetic resonance core analysis system (MesoMR23–060H–I, NiuMag, China) to obtain the *T*_2_ spectra, as shown in Figure 4. Before the experiments, the chemicals were dried at 120 °C until a constant weight was reached and then cooled to room temperature in a desiccator. In this study, crude oil and heavy water were used in the experiments. The aqueous solution was prepared by D_2_O, and the ionic composition is shown in Table 2. The rock cores were dried in the vacuum drying oven at 120 °C for 2 days, and the net weights were measured. Subsequently, the rock cores were vacuum-saturated in FW for approximately 50 h to ensure complete penetration of D_2_O into the pore spaces. The saturated cores were weighed using a high-precision electronic balance to calculate the pore volume and porosity. Simulated oil was prepared by blending the Chang 6 reservoir crude oil with kerosene, which led to a viscosity of 6 mPa·s. The simulated oil was then introduced to displace the core at 30 °C with a flow rate of 0.1 mL/min until no water was observed at the outlet. The confined water saturation was then calculated to be approximately 30.53%. The NMR experimental parameters were established as follows: wait time (TW) was set to 6000 ms, echo time (TE) was set to 0.254 ms, number of echoes (NECH) was set to 12,000, 90° pulse width (P1) was set to 5, and number of scans (NS) was set to 32. To simulate the waterflooding process, various injection fluids with different ionic compositions were injected into the rock cores at a rate of 0.1 mL/min. Each injection fluid was used to displace the cores until 5 pore volumes (PV) were injected, and the outlet water cut was approximately 100%. The *T*_2_ spectra of the rock cores and fluids produced during the displacement experiments with different ionic compositions were obtained using the NMR core analysis system.

## 3 Results

### 3.1 Wettability

**Figure 5:**
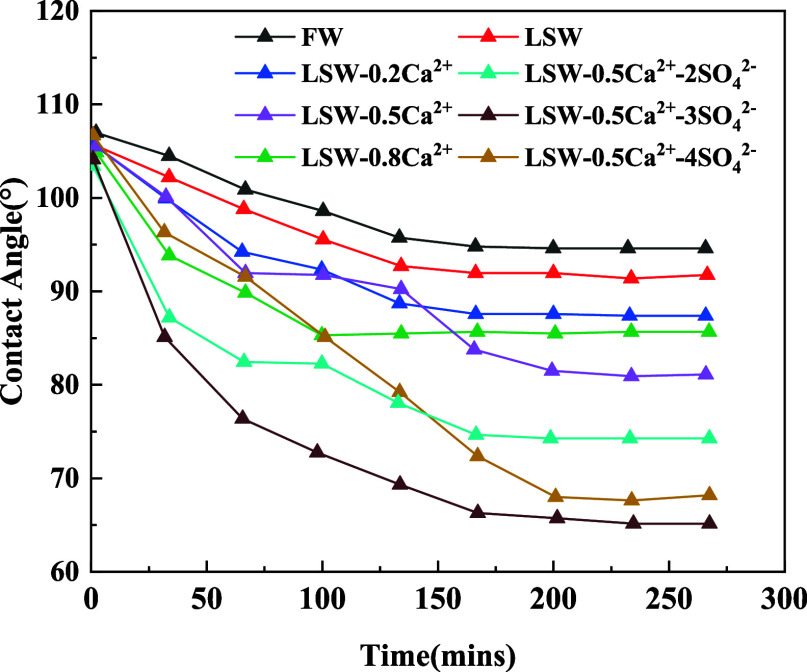
Contact angle in different injection aqueous solutions.

Figure 5 illustrates the variation of the oil droplet wetting angle with time on the rock surface in aqueous solutions of different ionic compositions. The results indicate that adjusting the mass concentrations of Ca^2+^ and SO_4_^2–^ in the injection water while maintaining the salinity of the injection water can effectively change the wettability of the rock surface. The oil droplets on rock surfaces in solutions FW, LSW, LSW-0.2Ca^2+^, LSW-0.5Ca^2+^, and LSW-0.8Ca^2+^ show contact angles of 94.60, 91.754, 87.38, 81.11, and 85.67°, respectively. In other words, as the concentration of Ca^2+^ in the solution decreases, the contact angle steadily decreases, indicating an improvement in the water wettability of the rock surface. The most favorable water wettability is observed in LSW-0.5Ca^2+^, which corresponds to the lowest Ca^2+^ ion composition. Further adjustments were made to the concentration of SO_4_^2–^ ions under specific conditions of the Ca^2+^ ion concentration. The contact angles of oil droplets on rock surfaces were measured in solutions LSW-0.5Ca^2+^-2SO_4_^2–^, LSW-0.5Ca^2+^-3SO_4_^2–^, and LSW-0.5Ca^2+^-4SO_4_^2–^ and stabilized at 74.26, 68.18, and 65.14° respectively. This indicates that as the SO_4_^2–^ concentration in the solution increases, the contact angle decreases, suggesting an improvement in the water wettability of the rock surface. In reservoirs with mixed wettability, it is generally accepted that the more water wettability of the reservoir, the more favorable the recovery.^33,34^ The contact angle of the crude oil on the rock surface in the LSW-0.5Ca^2+^-3SO_4_^2–^ water type is minimized, resulting in the strongest water wettability on the rock surface. The better water wettability is observed in LSW-0.5Ca^2+^-3SO_4_^2–^, which corresponds to the optimal composition of Ca^2+^ and SO_4_^2–^ ions.

### 3.2 Rock Core Displacement

#### 3.2.1 Oil Displacement Efficiency

**Figure 6:**
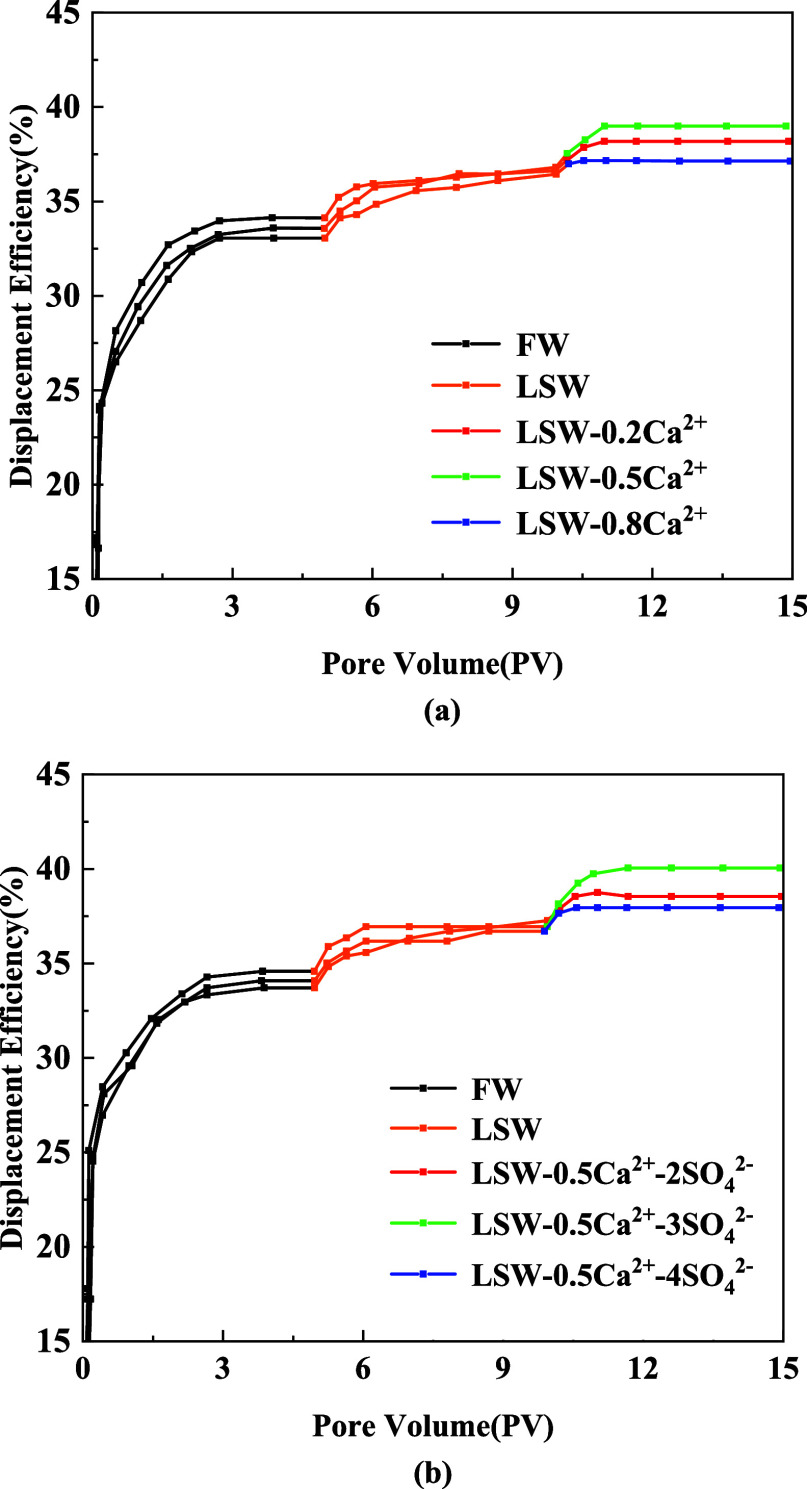
(a) Variation of oil displacement efficiency with different concentrations of Ca^2+^ in the solution; (b) variation of oil displacement efficiency with different concentrations of SO_4_^2–^ in the solution.

Figure 6a,b illustrates the curves showing the variations in oil recovery during the displacement process using different Ca^2+^ mass concentrations. It can be seen from Figure 6a that during the FW injection period, the oil recovery rates for the three cores stabilize at 33.57, 34.12, and 33.05%, respectively. After switching the injection water to LSW, the oil recovery rates increase and stabilize at 36.80, 36.62, and 36.44%, respectively. This study shows that switching to LSW injection water enhances oil recovery with increases of 3.23, 2.5, and 3.39%, respectively. Upon switching to injection waters with different Ca^2+^ ion concentrations, LSW-0.2Ca^2+^, LSW-0.5Ca^2+^, and LSW-0.8Ca^2+^, the oil recovery rates stabilize at 38.18, 38.99, and 37.14%, respectively. These results correspond to oil recovery improvements of 1.38, 2.37, and 0.7%, respectively. As the Ca^2+^ mass concentration in the injection water increases, the oil recovery initially increases and then decreases. It can be seen that LSW-0.5Ca^2+^ shows the most significant improvement in oil recovery, while further increases or decreases in the Ca^2+^ ion concentration in the injection water have a negative effect on oil recovery. Figure 6b illustrates the variation curves of the oil recovery based on the adjustment of the SO_4_^2–^ ion concentration in the injection water by using the optimum Ca^2+^ ion concentration. The figure shows that during the FW injection phase, the oil recovery rates for the three cores stabilize at 33.71, 34.58, and 34.08%, respectively. By changing the injection water to LSW, oil recovery rates increase and stabilize at 37.27, 36.94, and 36.70%, respectively. The results demonstrate that the oil recovery rates increased by 2.56, 2.36, and 2.62%, respectively. Then, when switching to injection waters with different SO_4_^2–^ ion concentrations, LSW-0.5Ca^2+^-2SO_4_^2–^, LSW-0.5Ca^2+^-3SO_4_^2–^, and LSW-0.5Ca^2+^-4SO_4_^2–^, the oil recovery rates stabilize at 38.55, 40.05, and 37.95%, respectively. These results correspond to oil recovery improvements of 1.28, 3.11, and 1.25%, respectively. As the concentration of SO_4_^2–^ ions in the injection water increases, the oil recovery initially increases and then decreases. It can be seen that LSW-0.5Ca^2+^-3SO_4_^2–^ shows the most significant improvement in oil recovery. Continually changing the SO_4_^2–^ concentration in the injection water, either increasing or decreasing, has a negative effect on oil recovery. In summary, decreasing the Ca^2+^ concentration and increasing the SO_4_^2–^ concentration in the injection water while maintaining its salinity have been found to significantly improve oil recovery in tight sandstone reservoirs. There is an optimum ion concentration that maximizes oil recovery.

#### 3.2.2 Surface Reactions on the Rock Surface

Table 4 displays the alterations in the pH values before and after the rock powder reacted with distinct injection solutions. From the table, it can be seen that adjusting the Ca^2+^ ion concentration in the solution results in pH variations. Before the reaction, the pH values of the FW, LSW, LSW-0.2Ca^2+^, LSW-0.5Ca^2+^, and LSW-0.8Ca^2+^ solutions are 6.42, 7.98, 8.26, 8.34, and 7.99, respectively, with the pH values changing as the Ca^2+^ ion concentration decreases. After being reacted with rock powder, the pH values in the supernatant of LSW, LSW-0.2Ca^2+^, LSW-0.5Ca^2+^, and LSW-0.8Ca^2+^ stabilize at 6.73, 8.05, 8.67, 8.95, and 7.92, respectively, while the pH value of the FW solution remains almost unchanged and the pH values of the other solutions increase. The concentrations of Ca^2+^ and SO_4_^2–^ ions in the FW solution remain almost unchanged, while in the other solutions, the concentrations of Ca^2+^ ions increase from the initial compositions of 682.681, 136.536, 341.340, and 546.144 mg/L to 724.611, 352.079, 624.746, and 716.923 mg/L. The concentration of SO_4_^2–^ ions increases from the original value of 117.054 mg/L to 145.351, 197.747, 169.706, and 155.657 mg/L, respectively. The changes in pH and ion concentrations indicate a water–rock reaction at the rock surface involving ion exchange. Specifically, injected water caused the desorption of Ca^2+^ and SO_4_^2–^ ions fixed on the rock surface. Moreover, there is an optimum concentration of Ca^2+^ that increases the desorption rate of divalent ions, leading to changes in pH.

**Table 4:** Concentrations of Ca^2+^ and SO_4_^2–^and pH Values in the Solution after Reaction

	before reaction			after reaction		
solutions	Ca^2+^ (mg/L)	SO_4_^2–^ (mg/L)	pH	Ca^2+^ (mg/L)	SO_4_^2–^ (mg/L)	pH
FW	8022.43	302.915	6.42	7953.912	356.781	6.73
LSW	682.681	117.054	7.98	724.611	145.351	8.05
LSW-0.2Ca^2+^	136.536	117.054	8.26	352.079	197.747	8.67
LSW-0.5Ca^2+^	341.340	117.054	8.34	624.746	169.706	8.95
LSW-0.8Ca^2+^	546.144	117.054	7.99	716.923	155.657	7.92
LSW-0.5Ca^2+^-2SO_4_^2–^	341.340	234.108	8.31	654.652	243.119	8.94
LSW-0.5Ca^2+^-3SO_4_^2–^	341.340	351.162	8.45	693.826	337.031	8.99
LSW-0.5Ca^2+^-4SO_4_^2–^	341.340	468.216	8.32	659.630	453.685	7.69

Altering the SO_4_^2–^ ion concentration in the injection water leads to pH variations, with a decrease of SO_4_^2–^ ion concentration resulting in a decrease in pH. After reacting with rock powder, the pH values for LSW-0.5Ca^2+^-2SO_4_^2–^, LSW-0.5Ca^2+^-3SO_4_^2–^, and LSW-0.5Ca^2+^-4SO_4_^2–^ solutions stabilize at 8.31, 8.45, and 8.32, respectively, with the pH values changing with decreasing SO_4_^2–^ ion concentration. After the reaction, the pH values stabilize at 8.94, 8.99, and 7.69, respectively. Simultaneously, the concentrations of Ca^2+^ and SO_4_^2–^ ions in the supernatant increase, with Ca^2+^ concentrations changing from the initial composition of 341.340 to 654.652, 693.826, and 659.630 mg/L and SO_4_^2–^ ion concentrations changing from the initial composition of 234.108, 351.162, and 468.216 to 243.119, 337.031, and 453.685 mg/L. The variations in pH and ion concentrations indicate a water–rock reaction and ion exchange at the rock surface. The SO_4_^2–^ions promote the desorption of Ca^2+^ ions from the rock surface.

### 3.3 Nuclear Magnetic Resonance

#### 3.3.1 Oil Displacement Efficiency

**Figure 7:**
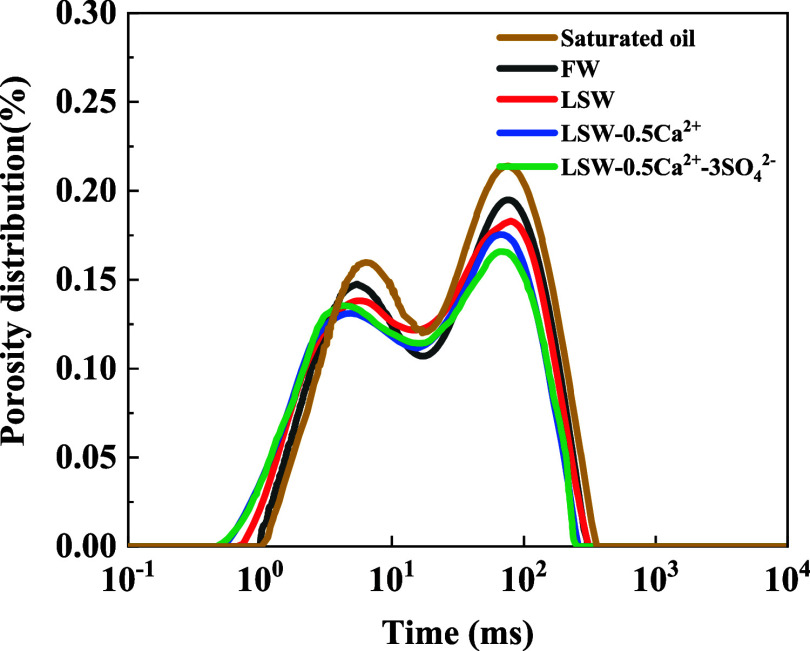
NMR *T*_2_ spectra of the rock core after oil displacement by injection of water with different ion compositions.

**Figure 8:**
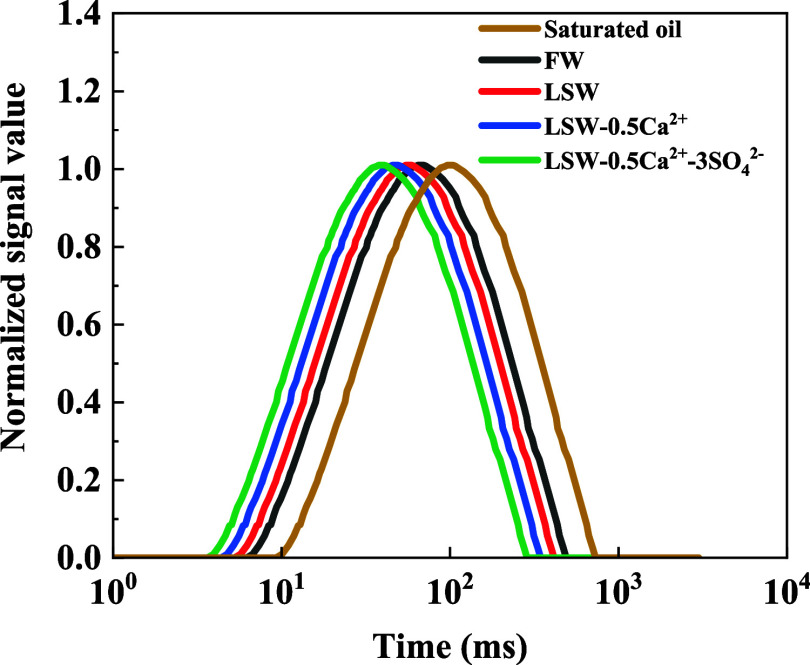
Normalized nuclear magnetic resonance (NMR) *T*_2_ spectrum curves of produced oil in water flooding with different ionic compositions.

During the water flooding experiments, heavy water (D_2_O) was utilized as the aqueous phase for water displacement oil examinations. Because the heavy water cannot contain hydrogen nuclei, the NMR *T*_2_ spectra cannot produce NMR signals, so the NMR *T*_2_ spectra are the *T*_2_ spectra of the oil phase in the pore space of the core. The *T*_2_ spectrum of the oil phase in the initial state before water injection reflects the saturation state of the oil in the pore space. It can be used to quantitatively analyze mobile oil in the initial saturation state of the core. Similarly, the *T*_2_ spectrum of the oil phase in the residual oil state after water flooding reflects the state and the amount of residual oil in the pore space after water flooding. Figure 7 displays the *T*_2_ spectra of cores that have been displaced by water with various ion compositions. Figure 8 displays the *T*_2_ spectra of the production oil after injection of different ion compositions into the core. The *T*_2_ spectra of the core in the initial saturated state indicate that there are double peaks on the left and right sides of the transverse relaxation time. The left peak corresponds to a transverse relaxation time of roughly 6 ms, while the right peak is at around 75.8 ms. These findings suggest that the reservoir rock has a high degree of development of small pore throats and strong inhomogeneity and belongs to the typical extra-low-permeability sandstone. After injection with water of different ionic compositions, the *T*_2_ spectra of the oil phase in different states changed significantly, where the left peak represents the residual oil content in the small pore space and the right peak represents the mobile oil content in the large pore space. After the injection of formation water (FW), a significant decrease in the peak values was observed on both sides. The remaining oil was concentrated in the large pore space and small pore space. By reducing the salinity of the injected water and increasing the mass concentration of SO_4_^2–^ and Ca^2+^, both the left peak and the right peak of the *T*_2_ spectra decreased. This indicates that the remaining oil in the pore space was mobilized after the injection. The recovery rate was calculated by determining the ratio of the curve envelope area of the *T*_2_ spectrum to the saturated oil curve, and the recovery rates of FW, LSW, LSW-0.2Ca^2+^, LSW-0.5Ca^2+^, and LSW-0.5Ca^2+^-3SO_4_^2–^ were 29.36, 34.32, 37.31, and 39.57%, respectively. The decrease in the *T*_2_ peak was found to be associated with changes in intrapore wettability and capillary force.^35,36^ Decreasing the salinity of the injected water and the mass concentration of Ca^2+^ and increasing the mass concentration of SO_4_^2–^ led to a more water-wettable rock surface, resulting in a thicker water film on the rock surface. Tight reservoirs have a low porosity and poor permeability, leading to greater resistance to fluid migration. In unconventional reservoirs, due to the micron- to nanometer-scale pores, the change of wettability and other interfacial properties can significantly influence the capillary pressure.^37^ As the reservoir core is a micron-scale pore network system, increasing the water film thickness caused some of the flow channels in the pore throat structure to decrease in size.^38^ This phenomenon strengthens the capillary force, resulting in the discharge of crude oil from the pore space and the reduction of the residual oil content.^38^

#### 3.3.2 Wettability Characteristics

According to the nuclear magnetic resonance relaxation mechanisms, the transverse relaxation time *T*_2_ is composed of surface, bulk, and diffusion relaxation processes.^39^ In a uniform magnetic field, the relaxation caused by diffusion can be disregarded, enabling the eq 1(39).
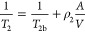
1

Therefore, within the rock cores, in addition to bulk relaxation, there is also surface relaxation of the oil adsorbed on the rock surfaces. The transverse relaxation time of the simulated oil in the produced fluid is the bulk transverse relaxation time and is the same as the remaining oil in the rock cores.^39^ In order to examine the changes in the surface transverse relaxation time of the rocks and investigate the changes in wettability, the geometric mean of the *T*_2_ spectra for the oil within the rock cores and the produced fluid were calculated according to eq 2.^40^ The geometric means of the *T*_2_ spectra calculated from Figures 6 and 7 are given in Table 5.
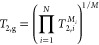
2

**Table 5:** Geometric Mean Values of *T*_2_ Spectra for Oil within Rock Cores and in the Produced Fluid

the geometric mean value of *T*_2_ spectra for oil within the rock cores/ms					the geometric mean value of *T*_2_ spectra for oil in the produced fluid/ms				
saturated oil	FW	LSW	LSW-0.5Ca^2+^	LSW-0.5Ca^2+^-3SO_4_^2–^	saturated oil	FW	LSW	LSW-0.5Ca^2+^	LSW-0.5Ca^2+^-3SO_4_^2–^
23.877	18.435	17.792	17.1031	16.7674	96.2165	64.1412	54.8397	45.9059	37.9795

When only oil and water are present in the pore space and the rock is mixed wet (eq 3), the *T*_2_ transverse relaxation time of the oil in the pore (eq 4) can be expressed separately according to eq 1(41)
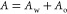
3
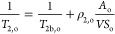
4

Looyestijn and Hofman^41^ found that the wettability of rock can be quantitatively characterized by the NMR wettability index
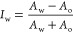
5

Because heavy water is used as the aqueous phase, only volume relaxation and surface relaxation of the crude oil exist in the rock cores. Therefore, the NMR wettability index calculation formulas for different displacement periods are given by eqs 1–6 as follows:^39^
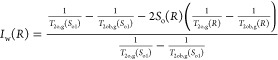
6

**Figure 9:**
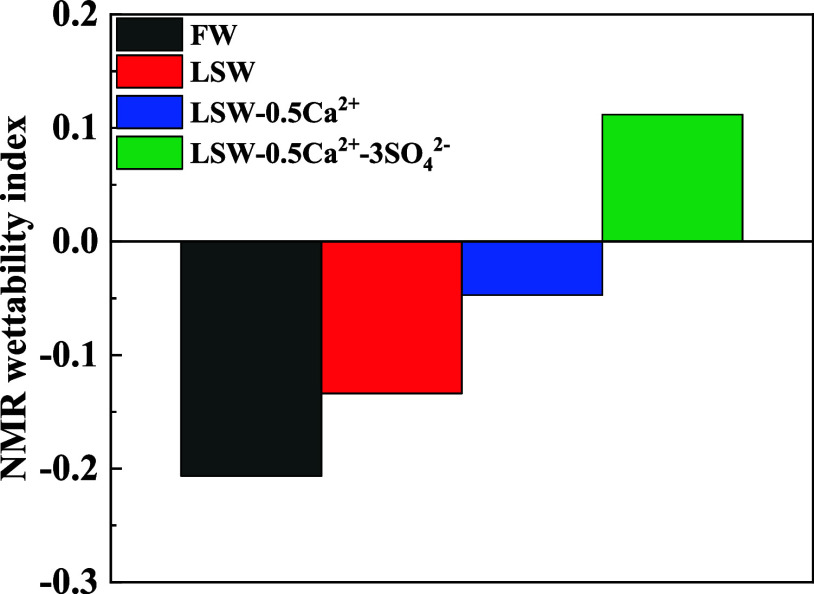
NMR wettability index of rock cores with different injection waters.

Figure 9 illustrates the NMR wettability index of rock cores subjected to displacement with different injection waters. It can be seen from the figure the wettability indexes of FW, LSW, LSW-0.5Ca^2+^, and LSW-0.5Ca^2+^-3SO_4_^2–^ are −0.206, −0.134, −0.047, and 0.112, respectively. The NMR wettability index ranges from [−1, 1], where [−1, −0.7] represents strong oil wetting, [−0.7, −0.3] represents oil wetting, [−0.3, −0.1] represents weak oil wetting, [−0.1, 0.1] represents neutral wetting, [0.1, 0.3] represents weak water wetting, [0.3, 0.7] represents water wetting, and [0.7, 1] represents strong water wetting.^39,42^ After ion adjustment, the wettability index of the rock cores increased gradually, from weakly oil-wet to weakly water-wet, indicating strengthened hydrophilicity at the rock surface.

## 4 Discussion

**Figure 10:**
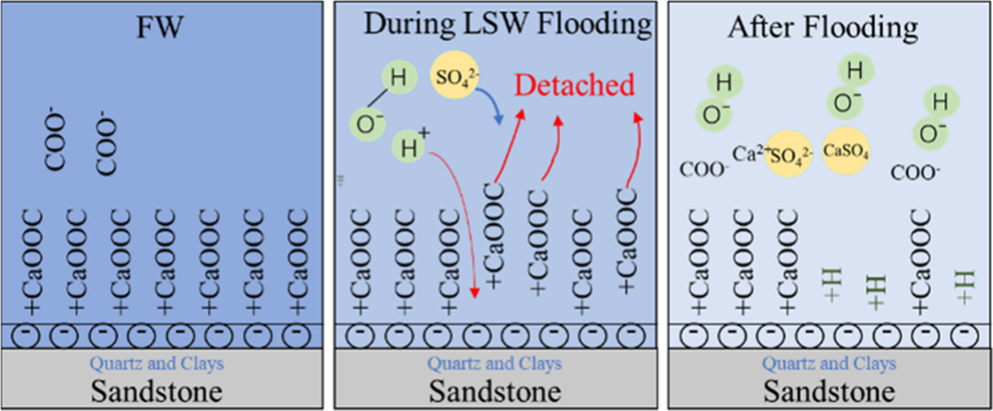
Mechanism of wettability alteration on the rock surface by low-salinity water.


The formation water contains numerous cations and anions in high concentrations such as Na^+^, Ca^2+^, Mg^2+^, Cl^–^, and SO_4_^2–^. The specific combinations and concentrations of these ions can significantly alter the wettability of the rock surface, thereby enhancing recovery. In the mixed wettability rock, oil molecules are tightly bound to the clay surface by various types of chemical bonds that promote the oil wettability of the rock surface.^43,44^ As shown in Figure 10, the sandstone surface (quartz and clay) and carboxylates are negatively charged. Divalent cations (Ca^2+^ ions) bind to carboxyl groups, producing −COOCa^+^, which tightly adsorbs the crude oil onto the rock surface.^45,46^ In the initial formation conditions, the high salinity formation water and crude oil are in equilibrium, and the crude oil is stably adsorbed on the rock surface. When the low-salinity water is injected into the formation, the equilibrium is broken.^45,46^ The electric double layer of the crude oil molecules and the rock surface swell.^45−47^ The monovalent cations (Na^+^) or protons (H^+^) in the water replace the Ca^2+^ involved in bridging on the rock surface, and the number of carboxylate groups bridged on the rock surface decreases. This replacement leads to a reduction in the number of carboxylate groups bridged to the rock surface. Consequently, the affinity of the rock surface for crude oil decreases, and the affinity for water molecules increases, resulting in the wettability of the rock surface being shifted toward water wettability. When the wettability changes, the crude oil molecules that were originally adsorbed on the rock surface become detached. This alteration in wettability results in a corresponding change in capillary pressure.^35,48^ Under the effect of the capillary force, the self-absorption phenomenon of hydrophilic rock can efficiently expel the crude oil from the pore space. The viscosity finger within the core is decreased, enhancing sweep efficiency and in turn, enhancing the recovery rate.^35,48^ When the divalent cation concentration in the water decreases, the polar components bridged to the surface of the rock become detached, resulting in water-wetness. This is demonstrated in ion exchange reactions 7 and 8,^45^ which are the processes of binding carboxylate groups to the rock surface and can also represent the process of detachment of carboxylate from the rock surface due to decreasing ion concentration.

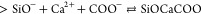
7
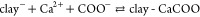
8

As shown in eq 7, the reaction proceeds to the left as the concentration of Ca^2+^ ions in the water decreases, and SiOCaCOO is transformed into SiO^–^, Ca^2+^, and COO^–^, which causes the carboxylic acid (COO^–^) to detach from the surface of the rock (SiO^–^), and the same is true for eq 8. The sulfate ion (SO_4_^2–^) carries the same electrical charge as the rock surface. As the concentration of sulfate ions (SO_4_^2–^) increases, they bond with Ca^2+^ ions to form complexes that prevent Ca^2+^ from bridging with the carboxylic acid group and the surface of the rock. This results in the desorption of crude oil from the rock surface and a reversal of wettability. Additionally, decreasing the concentration of Ca^2+^ ions and increasing SO_4_^2–^ ions in the injected water can lead to an increase in pH. At high pH levels, the rock minerals release hydroxyl ions (OH^–^) that chemically react with the reactive components of the crude oil to generate in situ surfactants.^49,50^ These reactions improve the water wettability of the rock.

## 5 Conclusions

1. Low-salinity water flooding is appropriate for tight sandstone reservoirs, as it can successfully enhance the wettability of rock surfaces by modifying the mass concentration of Ca^2+^ or SO_4_^2–^ ions.
2. Changing the mass concentration of Ca^2+^ ions or SO_4_^2–^ ions in the injection water can promote ion exchange on the rock surface, increase the pH, facilitate the detachment of adsorbed crude oil components from the rock surface, induce wettability changes, and improve oil recovery.
3. Adjusting the mass concentration of Ca^2+^ ions or SO_4_^2–^ ions in the injection water can change the wettability within the rock core, promote the expulsion of crude oil from small pores, and enhance oil recovery.

## Nomenclature

*K*_sp_: solubility product, mol^2^/L^2^
*T*_2_: transverse relaxation time, ms
*T*_2b_: transverse relaxation time of bulk fluid, ms
*T*_2,o_: transverse relaxation time of oil, ms
*T*_2b,o_: bulk transverse relaxation time of oil, ms
*T*_2,g_: geometric mean of the *T*_2_ spectrum, ms
*T*_2o,g_(*S*_o1_): geometric mean of *T*_2_ spectrum of oil in saturated core, ms
*T*_2bo,g_(*S*_o1_): geometric mean of bulk transverse relaxation time of oil in saturated core, ms
*T*_2o,g_(*R*): geometric mean of *T*_2_ spectrum of oil after different water injections, ms
*T*_2bo,g_(*R*): geometric mean of bulk transverse relaxation time of oil after different water injections, ms
ρ_2_: surface relaxivity, μm/ms
ρ_2o_: surface relaxivity of oil, μm/ms
*V*: volume of the pore, μm^3^
*A*: surface area of the pore, μm^2^
*A*_w_,*A*_o_: pore area wetted by water and oil, μm^2^
*I*_w_: NMR wettability index, nondimensional
*I*_w_(*R*): NMR wettability index for different water injections, nondimensional
*M*: total amplitude of all *T*_2_ spectra, nondimensional
*M*_*i*_: amplitude of the *i*-th *T*_2_ spectrum, nondimensional
*N*: total number of *T*_2_ spectra
*R*: different injected waters, nondimensional
*S*_w_,*S*_o_: saturation of water and oil, %
*S*_o_(*R*): oil saturation after different water injections, %
